# Multi-Omics Analysis of Fatty Acid Metabolism in Thyroid Carcinoma

**DOI:** 10.3389/fonc.2021.737127

**Published:** 2021-12-16

**Authors:** Jinghui Lu, Yankun Zhang, Min Sun, Changyuan Ding, Lei Zhang, Youzi Kong, Meng Cai, Paolo Miccoli, Chunhong Ma, Xuetian Yue

**Affiliations:** ^1^ Department of Hernia and Abdominal Wall Surgery, General Surgery, Qilu Hospital, Cheeloo College of Medicine, Shandong University, Jinan, China; ^2^ Key Laboratory for Experimental Teratology of Ministry of Education and Department of Immunology, School of Basic Medical Science, Cheeloo College of Medicine, Shandong University, Jinan, China; ^3^ Laboratory of Basic Medical Sciences, Qilu Hospital, Cheeloo College of Medicine, Shandong University, Jinan, China; ^4^ Department of Thyroid Surgery, General Surgery, Qilu Hospital, Cheeloo College of Medicine, Shandong University, Jinan, China; ^5^ Department of Obstetrics, The Second Hospital, Cheeloo College of Medicine, Shandong University, Ji’nan, China; ^6^ Key Laboratory for Experimental Teratology of Ministry of Education and Department of Cell Biology, School of Basic Medical Science, Cheeloo College of Medicine, Shandong University, Jinan, China; ^7^ Department of Ultrasound, Shandong Provincial Hospital Affiliated to Shandong First Medical University, Jinan, China; ^8^ Department of Surgery, University of Pisa, Pisa, Italy

**Keywords:** fatty acid metabolism, multi-omics analysis, thyroid carcinoma, therapeutic target, FATP2, CPT1A, LPL

## Abstract

**Objective:**

Papillary thyroid carcinoma (PTC) accounts for the majority of thyroid cancer and affects a large number of individuals. The pathogenesis of PTC has not been completely elucidated thus far. Metabolic reprogramming is a common feature in tumours. Our previous research revealed the reprogramming of lipid metabolism in PTC. Further studies on lipid metabolism reprogramming may help elucidate the pathogenesis of PTC.

**Methods:**

Clinical samples of PTC and para-tumour tissue were analysed using lipidomic, proteomic, and metabolomic approaches. A multi-omics integrative strategy was adopted to identify the important pathways in PTC. The findings were further confirmed using western blotting, tissue microarray, bioinformatics, and cell migration assays.

**Results:**

Multi-omics data and the results of integrated analysis revealed that the three steps of fatty acid metabolism (hydrolysis, transportation, and oxidation) were significantly enhanced in PTC. Especially, the expression levels of LPL, FATP2, and CPT1A, three key enzymes in the respective steps, were elevated in PTC. Moreover, LPL, FATP2 and CPT1A expression was associated with the TNM stage, lymph node metastasis of PTC. Moreover, high levels of FATP2 and CPT1A contributed to poor prognosis of PTC. In addition, ectopic overexpression of LPL, FATP2 and CPT1A can each promote the migration of thyroid cancer cells.

**Conclusions:**

Our data suggested that enhanced fatty acid metabolism supplied additional energy and substrates for PTC progression. This may help elucidating the underlying mechanism of PTC pathogenesis and identifying the potential therapeutic targets for PTC.

## Introduction

The incidence of thyroid carcinoma has increased rapidly in recent years. Papillary thyroid carcinoma (PTC) accounts for 80% of all cases of thyroid malignancies (1, 2). Although patients with PTC exhibit relatively good prognosis and low mortality after standard treatment, the recurrence rate is considerably high at 20%–30%, or even higher, in some subtypes of PTC (3). Additionally, some patients may die owing to their inability to undergo surgery or iodine-131 treatment at advanced tumour stages. In addition, there are no effective preventive methods or medical resources that can be used. To date, the pathogenesis of PTC remains unclear and can be confirmed only *via* thorough investigation.

Metabolic reprogramming is a common feature of tumours. Among them, lipid metabolism reprogramming is one of the most important metabolic reprogramming. Fatty acids (FAs) are essential components of lipids, such as triglycerides (TGs), phospholipids, glycolipids, cholesterol, and their esters. Additionally, FAs are essential components of membrane lipids and signal molecules and important substrates for energy metabolism (4). Increasing evidence shows that ovary, lung, stomach, colon, prostate, and breast cancer cells exhibit FA metabolism reprogramming, which is characterised by abnormally active FA synthesis and/or beta-oxidation (5, 6). Animal models and clinical studies have shown that the inhibition of key enzymes of FA synthesis or beta-oxidation, such as acetyl-CoA carboxylase, acetyl-CoA synthetase, ATP citrate lyase and carnitinepalmitoyl transferase 1C, could inhibit the growth of tumour cells and delay disease progression (7–11), suggesting that FA metabolism is necessary for tumour growth and proliferation and plays an important role in tumour development. Some authors, including us, have reported that FA levels were changed in PTC specimens (12–15), indicating the potential involvement of FA metabolism in PTC development. However, to date, only limited information has been obtained on FA reprogramming in PTC.

In this study, we performed multi-omics analysis of clinical samples and confirmed the expression patterns of key metabolic enzymes using omics analysis. We also evaluated the characteristics of FA metabolism in PTC, which may help clarifying the pathogenesis of PTC.

## Materials and Methods

### Ethics

This prospective study was approved by the institutional ethical committee of Qilu Hospital of Shandong University, Shandong Province, China. All the specimens were collected in the Qilu Hospital of Shandong University. The participants were provided with written informed consent for research.

### Participant Selection and Specimen Collection

Till October 2019, 48 consecutive patients (39 females and 9 males, age: 45.1 ± 8.5 years) who underwent total thyroidectomy or lobectomy and lymphadenectomy owing to thyroid nodule(s) detection or suspected PTC were included in this study. Forty-three patients were diagnosed preoperatively or suspected with malignancy, as observed using fine needle aspiration (FNA) according to the Bethesda classification. The FNA procedure was unsuccessful or rejected in the remaining five patients. Hence, the preoperative diagnosis was based on the analysis of ultrasound, computed tomography, and nuclear magnetic resonance imaging. Nine participants with diabetes, hyperlipidaemia, hypertension, anaphylactic diseases, or other metabolic diseases, or had a history of thyroid surgery or hormone treatment, were excluded from the study. Samples of nodule tissue and adjacent tissue were collected successfully from 32 patients during the surgical procedures. Whereas, seven patient’s specimens that the size of the nodule was too small to do subsequent histopathological examination and five patient’s specimens that based on the histopathological results were excluded for the following analyses. The tissue samples were frozen in liquid nitrogen immediately after harvesting during surgery and stored at -80°C until proteomics, lipidomics, and metabolomics analyses.

The patient characteristics are outlined in **Supplementary Table 1**.

### Lipidomic Analysis

The methyl tert-butyl ether (MTBE) method was used for lipid extraction. Briefly, the tissues were freeze-dried and weighed. Two hundred microliters of pre-chilled 75% methanol was added, and the suspension was sonicated for 10 min. Next, 500 μL of MTBE was added, the mixture was shaken for 1 h, and 125 μL water was added to separate the extraction solvent. After centrifugation at 16200 ×g at 4°C for 15 min, 400 μL of the supernatant was removed and dried under nitrogen. The residue was reconstituted with 120 μL of chloroform: methanol (v:v = 2:1), and 80 μL of the supernatant was placed in the sample vial for analysis. Details of the experimental procedure are provided in the **Supplementary Methods**. Principal component analysis (PCA), an unsupervised multivariate statistical model, and the supervised Partial Least Squares Discriminant Analysis (PLS-DA) model were used to explore the differences in lipid metabolism between the PTC and para-tumour groups. The parameters of PLS-DA model were shown in **Supplementary Table 2**.

### Proteomic Analysis

Samples from 27 patients were dispatched for analysis. Ten milligrams of each thyroid tissue specimen from each group were collected randomly, and nine samples were randomly pooled into one sample in each group. The samples from each group underwent the same pre-processing step and were labelled with 126C, 127C, and 128C tags for the PTC group and 129C, 130C, and 131C tags for the para-tumour group. The specific experimental procedure is described in the **Supplementary Methods**. Last, ANOVA was used for calculating the peptide and protein abundance. Normalisation was performed by averaging the abundances of all peptides. Medians were used for averaging. Differentially expressed proteins (DEPs) were filtered if they contained at least two unique peptides and showed over two-fold change, with *p* < 0.05.

### Metabolomic Analysis

Thyroid tissues were freeze-dried and weighed. Next, 150 μL of methanol was added to each sample, and the mixture was vortexed for 1 min and centrifuged at 16200 ×g at 4°C for 15 min. Next, 100 μL of the supernatant was added to a sample vial for subsequent analysis. Details of the procedure are provided in the **Supplementary Methods**. PCA and PLS-DA models were used to explore the differential metabolites between the PTC and para-tumour groups. The parameters of PLS-DA model were shown in **Supplement Table S2**.

### Cell Line, Plasmids, and Reagents

Methanol, acetonitrile, and isopropanol of HPLC grade were purchased from Merck (Darmstadt, Germany). Formic acid was obtained from Sigma-Aldrich (St. Louis, MO, USA). HPLC grade MTBE was purchased from Sinopharm Chemical Reagent Co., Ltd. (Shanghai, China). A Tandem Mass Tag (TMT) kit was purchased from Thermo Fisher Scientific Co. Ltd. (Waltham, MA, USA). Ultrapure water was prepared using a Milli-Q water purification system (Millipore Corp., Billerica, MA, USA).

BHP10-3 was gained from Qilu Hospital and were cultured in RPMI 1640 (Gibco) with 10% FBS (Gibco) at 37 °C.

Plasmids (pEnCMV-FATP2-flag, pEnCMV-CPT1A-flag, and pcDNA-LPL-HA) were purchased from Miaoling Biological Technology Co. LTD (Wuhan, China).

### Western Blot Experiment

Paired PTC and para-tumour tissues were homogenised in cell lysis buffer (Beyotime, P0013), and the protein extracts were analysed using a BCA protein assay kit (Beyotime, P0009). Equal quantities of proteins were separated using SDS-PAGE, transferred to polyvinylidene fluoride membranes (Thermo Fisher), and treated overnight with mouse monoclonal anti-actin (Abcam Cat# ab179467, RRID: AB_2737344), rabbit anti-LPL (Abcam, Cat# ab172953), rabbit anti-CPT1A (Proteintech Cat# 66039-1-Ig, RRID: AB_11041710), and rabbit anti-FATP2 (Proteintech Cat# 14048-1-AP, RRID: AB_2239416) antibodies as the primary antibodies at 4 °C. The membranes were washed three times with TBST and treated with goat Anti-Rabbit IgG H&L (HRP) antibody (Abcam Cat# ab205718, RRID: AB_2819160) or recombinant Anti-Mouse IgG antibody (Abcam Cat# ab190475, RRID: AB_2827162). FATP1 (Abcam Cat# ab69458, RRID : AB_1270734), FABP3 (Proteintech Cat# 60280-1-Ig, RRID : AB_2881398), FABP4 (Proteintech Cat# 67167-1-Ig, RRID : AB_2882463), FABP5 (Proteintech Cat# 66299-1-Ig, RRID : AB_2881682), CD36 (Proteintech Cat# 66395-1-Ig, RRID : AB_2811030), FABP1 (Proteintech Cat# 13626-1-AP, RRID : AB_2102017), ACADL (Proteintech Cat# 17526-1-AP, RRID : AB_2219661), ACADVL (Proteintech Cat# 14527-1-AP, RRID : AB_2288885), HADH (Proteintech Cat# 19828-1-AP, RRID : AB_10667408), HADHA (Proteintech Cat# 60250-1-Ig, RRID : AB_2881371), HADHB (Abcam Cat# ab110301, RRID : AB_10865743), ACAA2 (Abcam Cat# ab128911, RRID : AB_11143433). The signals were detected using TANON™ ECL chemiluminescence substrates with an automatic chemiluminescence/fluorescence image analysis system (Tanon, Shanghai).

### Immunohistochemistry

Tissue microarrays (HThyP120CS02) were purchased from Shanghai Outdo Biotech Company. Detailed information is available at the website (http://www.superchip.com.cn/biology/tissue.html). Each sample was differentially stained for lipoprotein lipase (LPL) (Abcam, ab172953, 1:100), FATP2 (Proteintech, 14048-1-AP, 1:200), and CPT1A (Proteintech, 14048-1-AP, 1:100). All primary antibodies were detected using Polymer-HRP, followed by DAB. All sections were counter stained with haematoxylin and dehydrated, and film-covers were mounted using Permount™ Mounting Medium. The results were analysed by at least three senior pathologists. The staining intensity was graded in four ranges (based on the intensity score). The positively stained PTC cells were categorised as follows (based on the percentage score): < 5% (0), 5%–25% (1), 26%–50% (2), 51%–75% (3), > 75% (4) (16). A final score of < 5 indicated low expression, whereas a score ≥ 5 indicated high expression.

### Molecular Network Analysis

To investigate the mechanism underlying PTC, the proteomic analysis results, metabolomics profile, and lipidomic profile were integrated using the Ingenuity Pathway Analysis (IPA, http://www.ingenuity.com) software. The *p* value and *z*-score are two important parameters in IPA. The *p* value, which is based on the right-tailed Fisher’s exact test algorithm, shows the probability between a set of meaningful molecules in an experiment, and the known process/pathway/transcription is derived from random matching. The *z*-score indicates the effect of molecular changes on biological processes. A *z-*score > 2 suggests that the corresponding pathways/networks are significantly activated, whereas a *z* score < -2 indicates that the corresponding pathways/networks are significantly inhibited.

### Bioinformatics Analysis

The expression of key genes (LPL, FATP2 and CPT1A) were analysed from Gene Expression Omnibus (GEO) database. Based on GEO dataset (GSE104006) (17), *LPL* (ID: ILMN_1786444) and *FATP2* (ID: ILMN_1700831) mRNA levels in thyroid cancer (n=29) were compared with normal tissues (n=5). *CPT1A* (203634_s_at) mRNA levels were analysed in GEO dataset (GSE65144) (18), which includes 12 thyroid cancer specimens and 13 normal tissues.

Survival analyses were performed with Kaplan-Meier Plotter (http://kmplot.com/analysis/) in a pan-cancer datasets (19), and thyroid carcinoma dataset was used for further analysis. The subjects were stratified by auto selected cut-off based on FATP2 or CPT1A expression. HR (Hazard Ratio) and *p* value were used to determine gene’s effect on thyroid cancer prognosis.

### Statistical Analysis

Data visualisation and statistical analysis (for data besides multi-omics and database data) were performed using GraphPad Prism version 8. *p* < 0.05 was considered significant for all experiments. Details of statistical tests for each experiment are provided in the figure legends. All data were presented in terms of mean ± S.D.

### Role of the Funding Source

The funding source played no role in the study design, data collection, analysis, and interpretation, writing of the report, or decision to submit the paper for publication. The corresponding author confirms he had full access to the data in the study and had final responsibility for the decision to submit for publication.

## Results

### Identification of Variant Lipids, Proteins, and Metabolites In PTC Specimens

**Figure 1:**
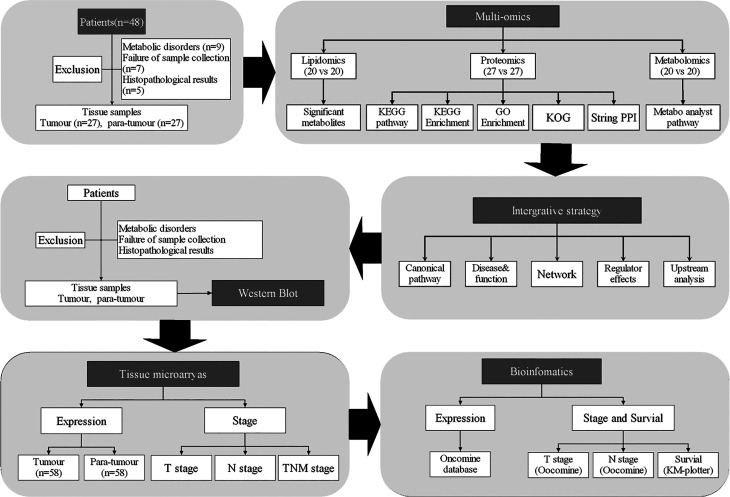
Flow chart of the study.

In order to evaluate lipid metabolism in thyroid cancer progression, we performed multi-omics analyses (**Figure 1**). Twenty paired specimens were used for the lipidomics and metabolomics analyses, and 27 paired specimens were used for proteomic analysis.

**Figure 2:**
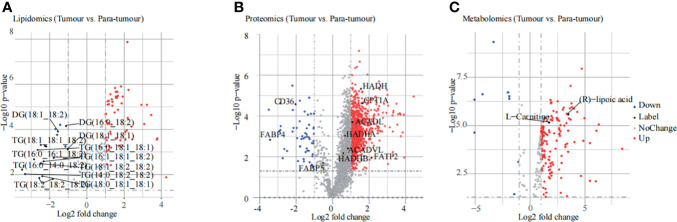
Results of multi-omics analysis. **(A, B)** The differentially expressed substances identified using lipidomic **(A)**, proteomic **(B)**, and metabonomic **(C)** analyses are displayed in the volcano plot. The red plots indicate an increase in expression, blue plots indicate a decrease in expression, grey plots indicate no change, and black plots indicate labelling.

Lipidomic analysis was performed using an untargeted lipid profiling platform based on Q Exactive mass spectrometry combined with the LipidSearch software for data pre-processing and lipid identification. The principal component analysis (PCA) score plot results showed that PTC and para-tumour groups were separated into two classes (**Supplementary Figures 1A, B**), indicating a notably different lipid metabolome between these two groups. These results were confirmed in the Partial Least Squares Discriminant Analysis (PLS-DA) score plot (**Supplementary Figures 1C, D**). Importantly, the differentially expressed lipids between the PTC and para-tumour groups were detected by combining variable importance in the projection (VIP) > 1 with *p <* 0.05 to select the differential levels of lipids (**Figure 2A**). Among the differentially expressed lipids, the TG and diacylglycerol (DG) contents decreased more obviously in the PTC group than in the para-tumour group. Meanwhile, the levels of phospholipids (phosphatidylcholine (PC), phosphatidylinositol (PI), phosphatidyl glycerol (PG), cephalin (PE), and sphingolipids (sphingomyelin (SM) and Cer), which are the primary components of biofilms and can be disintegrated into secondary messengers, increased by over two and three folds in the PTC group (**Figure 2A** and **Supplementary Figure 1E**). The evidence indicated that PTC was considerably influenced by lipid metabolism.

For proteomic analysis, PCA was used to explore the differences in protein expression between the PTC and para-tumour groups (**Supplementary Figure 2A**). The PCA results were confirmed using the PLS-DA score plot (**Supplementary Figure 2B**). In total, 2975 differentially expressed proteins (DEPs) were detected. Compared with the para-tumour group, in the PTC group, most proteins were up-regulated (fold change ≥ 2, *p* < 0.05), but several proteins were also down-regulated (fold change ≤ -2, *p* < 0.05), with false discovery rate (FDR) < 1% (**Figure 2B**). According to the threshold value of significant change as the demarcation, the data were primarily divided among three categories: the red point represents up-regulation, the blue point represents down-regulation, and the grey point represents no change. In particular, the fatty acid metabolism related genes, such as FATP2, ACADL, CPT1A, and so on, were elevated in PTC specimens compared to that in para-tumor specimens (**Figure 2B**). These data indicated that PTC progression was influenced by FA metabolism.

In the metabolomic analysis, the PCA and PLS-DA results indicated a clear distinction between the PTC and para-tumour groups. In addition, quality control (QC) samples were well clustered in the positive and negative ion modes, indicating good system stability (**Supplementary Figure 3**). Moreover, 217 endogenous substances with VIP > 1 and *p* < 0.05 were identified between the PTC and para-tumour groups (**Figure 2C**). (R)-lipoic acid and L-carnitine, which are strongly associated with FA oxidation, were present at high levels in the PTC group (**Figure 2C**). The pathway analysis module in MetaboAnalyst 3.5 was used to detect the major metabolic pathways involved in PTC progression. Several differential metabolic pathways were detected, such as the transfer of acetyl groups into the mitochondria, beta-oxidation of very-long-chain fatty acids (VLCFAs), and oxidation of branched chain FAs (**Supplementary Figure 4A**). These data also suggested that FA metabolism plays an important role in PTC development.

### Multi-Omics Integration Analysis Showed the Role of Lipid and FA Metabolism in PTC Progression

**Figure 3:**
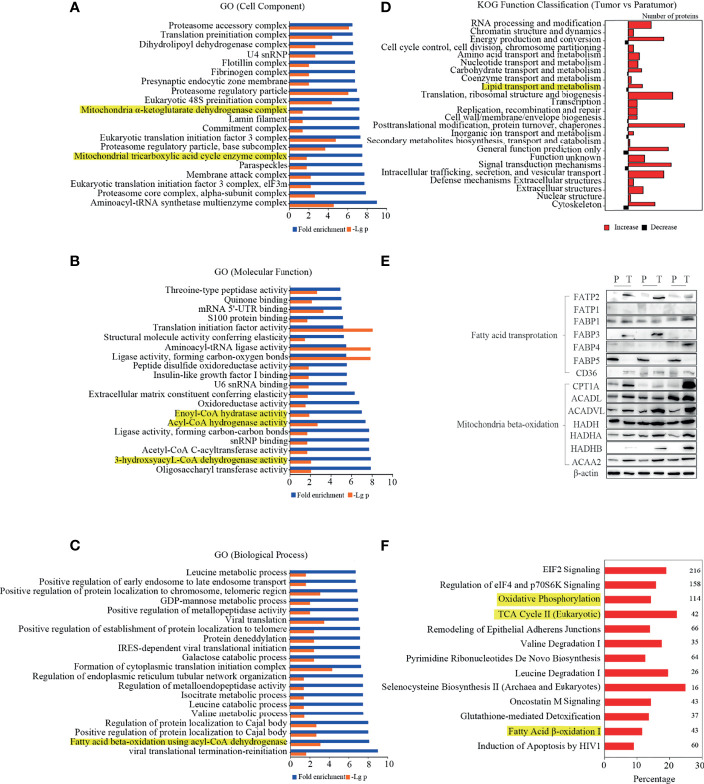
Differentially expressed proteins and pathways in PTC. **(A–D)** Gene ontology (GO) analysis and Eukaryotic Orthologous Groups (KOG) protein classifications were used to analyse the differentially expressed proteins. The lipid metabolism-related classes were highlighted in yellow. **(E)** The expression levels of key enzymes in fatty acid metabolism were determined in thyroid cancer and para-tumour specimens by western blot. **(F)** Upregulated canonical pathways exceeding the threshold [-log(p-value) ≥1.3, |Z score|≥2] were displayed in the figure. The numbers on the right indicate the count of genes.

To identify the lipid metabolism key proteins involving in PTC development, the proteomics data were used to profile the cellular component, biological process, molecular function, and KOG analysis. As shown in **Figure 3A**, mitochondrial proteins, which were differentially expressed in the PTC group, were enriched. The expression of enzymes involved in FA beta-oxidation, including enoyl-CoA hydratase, acyl-CoA dehydrogenase, and 3-hydroxyacyl-CoA dehydrogenase was enriched and related to molecular function (**Figure 3B**). The biological process analysis indicated that FA beta-oxidation with acyl-CoA dehydrogenase was significantly activated (**Figure 3C**). Furthermore, KOG analysis showed that lipid transport and metabolism were significantly enriched (**Figure 3D**). These data confirmed that lipid metabolism was associated with PTC. Importantly, heatmap showed that many fatty acids metabolism key enzymes were dysregulated in PTC (**Supplementary Figure 4B**). Then, the protein candidates involving in lipid metabolism were screened by western blot. As shown in **Figure 3E**, FATP2 and CPT1A were the significantly elevated protein in thyroid cancer specimens.

To confirm the above data, the IPA software was used for the integrative analysis of protein, metabolite, and lipid expression between PTC and para-tumour tissues. As shown in **Figure 3F**, multiple metabolic pathways were up-regulated in the PTC group compared to that in the para-tumour group. Interestingly, oxidative phosphorylation, TCA cycle II (eukaryotic), and FA beta-oxidation I, which are related to FA utilisation, were activated in the PTC group. The findings indicated that FA transport and oxidation were considerably activated in PTC.

### LPL Is Upregulated to Accelerate TG Hydrolysis in PTC

**Figure 4:**
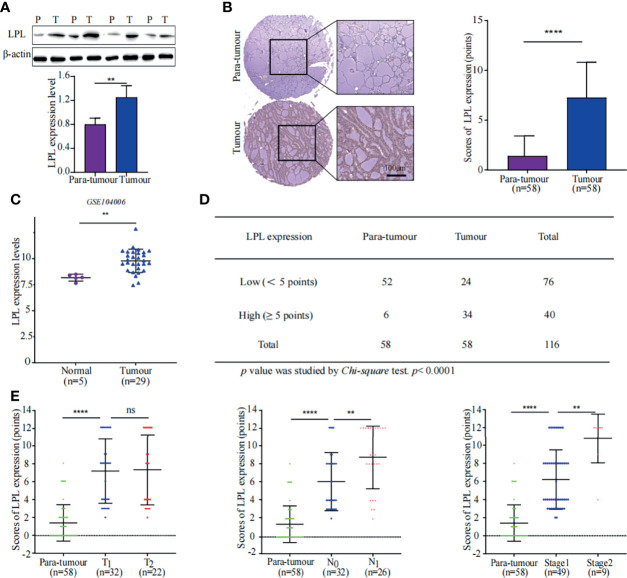
High expression of LPL was associated with poor prognosis in PTC. **(A)** LPL expression in tissue samples was measured using western blotting (WB). Representative WB diagrams of LPL expression are shown in the top panel. The quantification data are shown in the lower panel, **p < 0.01. The error bar indicates s.d., two-tailed unpaired *t*-test. P indicates para-tumour, T indicates tumour. **(B)** LPL expression and localisation were evaluated using tissue microarray. The left panel shows immunohistochemical staining for LPL (brown) with Mayer’s haematoxylin counterstain. Scale bar 100µm. Statistical data of LPL protein expression in 58 paired samples are shown in the right panel. ****p < 0.0001. Error bar indicates *s.d.*, two-tailed unpaired t-test. **(C)** Data of LPL mRNA levels were obtained from publically available dataset (GSE104006). **p < 0.01. The error bar indicates *s.d.*, student *t*-test. **(D)** Statistical analysis of LPL expression in tissue microarray. *Chi-square* test was used to analyse significant differences, ****p < 0.0001. **(E)** Correlation between LPL expression and stages of thyroid cancer. The graph in the left panel shows that LPL expression was positively correlated with the T stage in PTC. The graph in the middle panel shows that LPL expression was positively correlated with the N stage in PTC. The graph in the right panel shows that LPL expression was positively correlated with the TNM stage in PTC. ****p < 0.0001, **p < 0.01; ns indicates: not statistically significant. All error bars indicate *s.d*., two-tailed unpaired t-test.

Lipoprotein lipase (LPL) is the key enzyme in TG hydrolysis that catalyses TG into FAs. The proteomics data did not indicate the upregulation of LPL. Considering that the TG content was considerably low and proteomic analysis was semi quantitative, WB and IHC were performed to confirm LPL expression in the PTC and para-tumor specimens (**Figures 4A, B**). LPL was significantly upregulated in the PTC specimens than in adjacent tissues. Consistently, LPL expression elevation in thyroid cancer specimens were also observed in a GEO dataset (GSE104006) (**Figure 4C**) (17). Furthermore, immunohistochemical staining also revealed that the number of high-LPL specimens in the tumour group was more than that in the para-tumour group (**Figure 4D**). More importantly, LPL expression indicated the growth advantage and lymph node metastasis in PTC (**Figure 4E**). LPL up-regulation promotes TG hydrolysis, as indicated by the low TG levels in lipidomic analysis (**Figure 2A** and **Supplementary Figure 1E**). Pathway analysis also indicated that FA metabolism was considerably enhanced in PTC (**Figure 3F**). The above data indicates LPL plays a crucial role in thyroid progression by promoting FA metabolism.

### FATP2 Mediates FA Transport to Promote PTC

FA transport proteins (FATPs) are commonly known to facilitate the uptake of VLCFAs and long-chain FAs. FATP2, a FATPs family member, has been shown to mediate FA transport to damaged neutrophils to promote cancer (20). Complementation experiments in yeast showed that mouse FATP2 performs two functions: transport of exogenous VLCFAs across the plasma membrane and synthesis of PI (21–23). Proteomic analysis showed that the expression of FATP2, which is involved in the transport of exogenous FAs, was up-regulated 4.4-fold in PTC compared to that in para-tumour specimens (**Figure 2B**).

**Figure 5:**
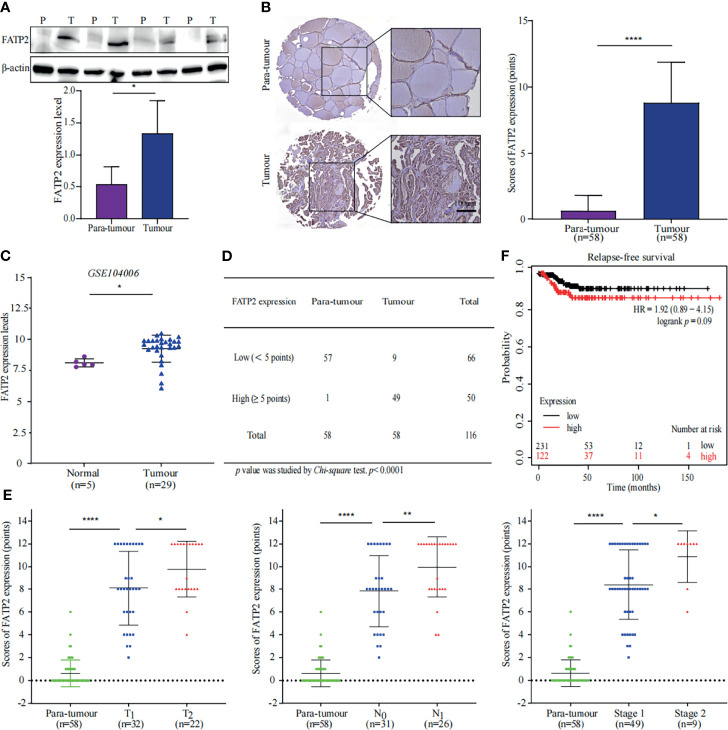
Thyroid carcinoma is promoted by FATP2 overexpression. **(A)** FATP2 expression in the tissue samples was measured using western blotting (WB). Representative WB diagrams of FATP2 are shown in the top panel. Quantification data are shown in the lower panel, *p < 0.05. Error bar indicates *s.d.*, two-tailed unpaired *t*-test. P indicates para-tumour, T indicates tumour. **(B)** FATP2 expression and localization in tissue microarray. Representative images of immunohistochemical staining for FATP2 (brown) with Mayer’s haematoxylin counterstain are shown in the left panel. Scale bar 100µm. Statistical data of FATP2 protein levels in 58 paired thyroid samples are shown in the right panel. ****p < 0.0001. Error bar indicates *s.d.*, two-tailed unpaired *t*-test. **(C)** Data of FATP2 mRNA levels were obtained from publicly available dataset (GSE104006). *p < 0.05. The error bar indicates *s.d.*, student *t*-test. **(D)** The number of FATP2-positive tissue samples in the tissue microarray. The *Chi-square* test was used to analyse the significant difference, ****p < 0.0001. **(E)** Correlation between FATP2 expression and thyroid cancer progression. The graph in the left panel shows that FATP2 expression was positively correlated with the T stage of PTC. The graph in the middle panel shows that FATP2 expression was positively correlated with the N stage of PTC. The graph in the right panel shows that FATP2 expression was positively correlated with the TNM stage of PTC. ****p < 0.0001, **p < 0.01, *p < 0.05. All error bars indicate *s.d.*, two-tailed unpaired *t*-test. **(F)** High FATP2 expression was associated with poor relapse-free survival. The online tool KM plot (http://kmplot.com/analysis/) was used to analyse thyroid carcinoma in pan-cancer datasets.

Further WB and IHC were performed to confirm our findings. A similar trend of FATP2 up-regulation in thyroid cancer specimens were observed (**Figures 5A, B**). Consistently, increasing of FATP2 expression was displayed in an online dataset (GSE104006) (**Figure 5C**) (17). Furthermore, immunohistochemical staining was performed using tissue microarrays and peritoneal modules. The number of tumour tissues with high FATP2 expression scores was more than the number of para-tumour tissues with high FATP2 expression scores (**Figure 5D**). FATP2 expression was significantly different (*p* value < 0.0001) between the tumour and para-tumour groups and was positively correlated with tumour size, lymph node metastasis, and TNM stage (**Figure 5E**). Importantly, patients with low FATP2 (SLC27A2) expression could achieve better relapse-free survival (KM plot) than patients with higher expression (**Figure 5F**) (19). These data indicate FATP2 promotes PTC growth and metastasis, which is correlated with poor prognosis.

### CPT1A Expression Favours Active Tumour FA Beta-Oxidation in PTC

**Figure 6:**
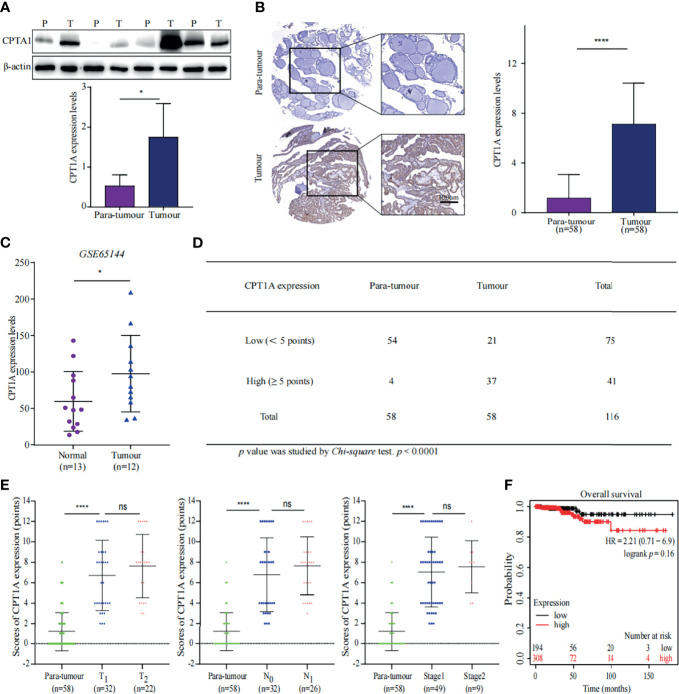
CPT1A expression favours PTC progression. **(A)** CPT1A expression in tissue samples was measured using western blotting (WB). Representative WB diagrams of CPT1A are shown in the top panel. The quantification data are shown in the lower panel, *p < 0.05. Error bar indicates *s.d.*, two-tailed unpaired *t*-test. P indicates para-tumour, T indicates tumour. **(B)** CPT1A expression and localisation in the tissue microarray. Representative images of CPT1A (brown) immunohistochemistry in the tissue microarray are shown in the left panel. Scale bar 100µm. Statistical analysis of CPT1A protein levels in 58 paired tissue samples is shown in the right panel. ****p < 0.0001. Error bar indicates *s.d.*, two-tailed unpaired *t*-test. **(C)** Data of CPT1A mRNA levels were obtained from publicly available dataset (GSE65144). *p < 0.05. The error bar indicates *s.d.*, student *t*-test. **(D)** The number of high-CPT1A expression samples in the tissue microarray. The P value was obtained using the *Chi-square* test to analyse the significant difference, ****p < 0.0001. **(E)** Relationship between CPT1A expression and stages of thyroid cancer. Data shown in the graphs for CPT1A expression were not discrepant regarding T stage, N stage, or TNM stage of PTC, ****p < 0.0001; ns indicates: not statistically significant. All error bars indicate *s.d.*, two-tailed unpaired *t*-test. **(F)** High levels of CPT1A were associated with poor overall survival by analysing a pan-cancer datasets through KM plot (http://kmplot.com/analysis/).

Proteomic analysis showed that the key enzymes of FA beta-oxidation were upregulated, which indicated that FA beta-oxidation was enhanced in PTC. Carnitine palmitoyltransferase 1A (CPT1A) is a critical enzyme in FA beta-oxidation. Indeed, the proteomics data showed CPT1A was increased in the PTC group (**Figure 2B**). This prompted us to further evaluate the protein expression of CPT1A in patient specimens. First, we performed WB and IHC to determine expression of CPT1A in tumour and para-tumour tissues. The results showed CPT1A was overexpressed in PTC specimens (**Figures 6A, B**). The findings were confirmed using a GEO dataset (GSE65144) (**Figure 6C**) (18). Together, these data confirmed the significant up-regulation of CPT1A in PTC.

We further analysed the immunohistochemical staining results of the tissue microarray. The PTC specimens received a greater number of high scores than the para-tumour specimens (**Figure 6D**). Although, CPT1A expression was not proportional with tumour size or lymph node metastasis (**Figure 6E**), high expression of CPT1A was negatively associated with overall survival (OS) of patients with thyroid cancer (**Figure 6F**) (19). Above all, these findings suggested CPT1A is a poor prognostic factor for PTC, and CPT1A suppression may help prevent poor prognosis in patients with PTC.

### Reprogrammed FA Metabolism Plays a Dominant Role in PTC

**Figure 7:**
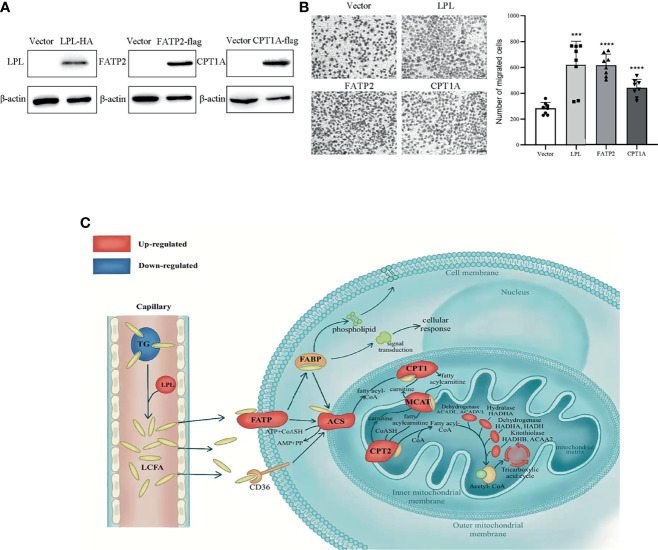
Activated lipid metabolism in thyroid carcinoma. **(A)** Overexpression of LPL, FATP2 and CPT1A in BHP10-3 cells were confirmed by western blot. **(B)** Cell migration was examined in BHP10-3 cells with LPL, FATP2 and CPT1A overexpression. ***p < 0.001, ****p < 0.0001. All error bars indicate *s.d.*, two-tailed unpaired *t*-test. **(C)** As shown, long-chain fatty acids (LCFAs) are released from triglycerides (TGs) by lipoprotein lipid (LPL) and transported to cells by fatty acid transport protein 2 (FATP2). Carnitine palmitoyltransferase 1A (CPT1A) controls the utilisation of LCFA for energy supply. Furthermore, LCFAs can be used for cell construction and signal transduction. Red indicates up-regulation and blue indicates down-regulation. TG, triglyceride; FABP, fatty acid binding protein; ACS, Acyl-coenzyme A synthetase; CPT2, Carnitine palmitoyltransferase 2; MCAT, mitochondria carnitine acylcarnitine translocase.

Metastasis is the major obstacle for thyroid cancer treatment. In order to verify that LPL, FATP2 and CPT1A involve in metastasis of thyroid cancer, these genes were overexpressed in BHP10-3 cells. As shown in **Figure 7A**, LPL, FATP2 and CPT1A protein levels were enhanced in overexpression vectors transfected BHP10-3 cells, respectively. Interestingly, the migrated cells numbers were significantly increased in LPL, FATP2 and CPT1A transfected BHP10-3 cells compared to their control vectors transfected cells (**Figure 7B**). Together, LPL, FATP2 and CPT1A activate fatty acid metabolism to promote thyroid cancer progression.

To our knowledge, this is the first study to show the complete changes in fatty acid metabolic pathways in PTC, as depicted in **Figure 7C**, using integrated analysis. The findings indicated that the levels of TG, one of the most important forms of stored FAs in the body, were considerably decreased. The FAs, hydrolyse from TGs by LPL, enter cells *via* FATP2 and transport into the mitochondria for beta-oxidation *via* CPT1A. Besides, FAs converted to phospholipids serve as cell membrane components (PE, PC, and PI) and disintegrated into secondary messengers for signal transduction pathways. The findings indicate that the metabolic reprogramming of FAs was enhanced and played a crucial role in PTC.

## Discussion

Morbidity owing to thyroid carcinoma has increased in recent times, and early metastasis and relapse have been reported (24). Treatment for papillary thyroid carcinoma (PTC) does not include advanced therapy beyond surgery and iodine-131 administration. Surgical interventions can pose a serious burden on patients from economic and psychological perspectives. Therefore, there is an urgent need for determining the mechanism underlying PTC progression and identifying potential targets for clinical treatment. Multi-omics analysis has been used to explore the progression of high-grade bladder cancer as well as to delineate opportunities for therapeutic and prognostic intervention (25). To our knowledge, ours is the first study to focus on the lipid mechanism underlying PTC progression with integrated lipidomics, proteomics, and metabolomics approaches.

Metabolic reprogramming is a prerequisite for the rapid proliferation of cancer cells. In the past few years, studies on the processes of lipid metabolism, such as lipid biosynthesis, transportation, and beta-oxidation, have received increasing attention owing to their potential involvement in tumour progression (26). Consistent with these findings, the data of our integrated analysis showed that fatty acid (FA) transportation and beta-oxidation were enhanced in thyroid carcinoma. Lipids supply the primary structural FAs and glycerides of biological plasma membrane, and provide potential energy substrates, which support rapid tumour proliferation (27). Previously, we have demonstrated that the levels of FAs were significantly low in patients with thyroid carcinoma (13, 14). These initial studies served as an indication for this work. Accordingly, we performed multi-omics analysis to evaluate the importance of lipid metabolism in thyroid carcinoma. We found that the phospholipid and cholesterol content were higher, and the TG and DG contents were lower in the PTC group. In the PTC group, certain FA metabolism key enzymes were overexpressed, and the level of intermediate products of FA utilisation was higher. These indicated that as primary components of the cytomembrane, phospholipids and cholesterol were associated with the rapid and uncontrolled proliferation of tumours. Triglyceride (TG) and diglyceride (DG) may contribute to tumour progression by acting as FA suppliers. Above all, FA metabolism plays a critical role in PTC development.

Lipoprotein lipase (LPL) catalyses the hydrolysis of TG in chylomicrons and very-low-density lipoproteins, liberates FAs from TGs, and increases the cellular uptake of lipoproteins in a non-catalytic manner (28). Following this, FAs are transported into cells as a part of phospholipids and/or beta-oxidation substrates. LPL is primarily expressed in adipose, skeletal muscle, and heart cells (29). Mounting evidence showed that LPL-mediated extracellular lipolysis occurs in breast cancer, liposarcomas and liver steatosis, which expression was considered to increase the free FA content (30, 31). Recently, studies have implicated LPL as a biomarker for poor prognosis in chronic lymphocytic leukaemia (32, 33). Therefore, LPL promotes the proliferation of certain types of cancer cells. Here, our findings indicated the upregulation of LPL in PTC and the direct correlation of LPL expression with tumour size and lymph node metastasis. This suggested that LPL was one initiator that supplied FAs as substrates of energy metabolism and structural components in PTC, and LPL was a poor prognostic factor for PTC.

Fatty acid transport proteins (FATPs) are members of the FA receptor family (34). Our study showed that FATP2 was upregulated and played a critical role in PTC. FATP2 performs a dual function, facilitating the transport of exogenous FA and activating PI synthesis (21–23, 35). FATP2 was up-regulated in melanoma cells and was a poor prognostic factor for melanoma (36). Our findings showed that lipid transportation was enhanced and FATP2 was overexpressed in PTC tissues. WB, IHC, and bioinformatics analysis indicated that FATP2 was a poor prognostic factor for thyroid tumour progression. These findings indicated that FATP2 captured and transported FAs to PTC cells for thyroid cancer progression.

Carnitine palmitoyltransferase 1A (CPT1A) is a member of CPT1 family, which controls the rate-limiting step of FA oxidation. Previous studies have shown that FAs are transported through the mitochondrial membrane by CPT1A, which could form a complex with long chain acyl-CoA synthetase and voltage-dependent anion channel on the mitochondrial outer membrane (37). CPT1A overexpression was associated with poor OS in patients with ovarian cancer (38). CPT1A was shown to facilitate the trafficking of FAs between the mitochondria and cytoplasm, which promoted FA utilisation in the acceleration of nasopharyngeal carcinoma (39, 40). Here, multi-omics analysis showed that CPT1A, the rate limiting enzyme in FA oxidation, was up-regulated in PTC, and high CPT1A expression was related to poor patient survival. Our data suggested that CPT1A expression could influence FA metabolism in PTC. Thus far, CPT1A could be a suitable therapeutic target for thyroid cancer.

Lipid metabolism has garnered considerable attention ever since it was significantly unregulated in cancers. However, the mechanism underlying lipid metabolism reprogramming in PTC is yet to be clarified. In this study, lipidomic analysis showed that the lipid levels differed between para-tumour and PTC tissues. The multi-omics analysis, experimental findings, and bioinformatics data indicated that FA transportation and beta-oxidation were significantly enhanced in PTC. These findings provide insights on the mechanism underlying PTC progression and may help identify a target for preventing tumour initiation and progression and improving prognosis.

## Data Availability Statement

The datasets presented in this study can be found in online repositories. The names of the repository/repositories and accession number(s) can be found below: PRIDE, PXD027538.

## Ethics Statement

The studies involving human participants were reviewed and approved by the Institutional ethical committee of Qilu Hospital of Shandong University, Shandong Province, China. The patients/participants provided their written informed consent to participate in this study. Written informed consent was obtained from the individual(s) for the publication of any potentially identifiable images or data included in this article.

## Author Contributions

Literature search and study design: JL, CM, and PM. Data collection, analysis: JL and XY. Sample collection: CD. Experiment: YZ, MS, YK, and MC. Manuscript writing: JL and MS. Paper review: JL, XY, and CM. Figure drawing: LZ. All authors contributed to the article and approved the submitted version.

## Funding

This work was supported by National Natural Science Foundation of China (No. 81702647, NO. 81972819), Taishan Scholar Foundation of Shandong Province (No. tspd20181201), Collaborative Innovation Center of Technology and Equipment for Biological Diagnosis and Therapy in Universities of Shandong, Natural Science Foundation of Shandong Province (No. ZR201702190483, No. ZR2020YQ57), and Key Technology Research and Development Program of Shandong (No. 2016GSF201161).

## Conflict of Interest

The authors declare that the research was conducted in the absence of any commercial or financial relationships that could be construed as a potential conflict of interest.

## Publisher’s Note

All claims expressed in this article are solely those of the authors and do not necessarily represent those of their affiliated organizations, or those of the publisher, the editors and the reviewers. Any product that may be evaluated in this article, or claim that may be made by its manufacturer, is not guaranteed or endorsed by the publisher.

## Abbreviations

PTC, papillary thyroid carcinoma, FNA, fine needle aspiration, FA, fatty acid, TG, triglyceride, DG, diacylglycerol, FDR, false discovery rate, QC, quality control, LPL, lipoprotein lipase, FATP2, fatty acid transport protein 2, CPT1A, carnitine palmitoyltransferase 1A, FABP, fatty acid binding protein, PCA, principal component analysis, PLS-DA, partial least squares discriminant analysis, OS, overall-survival. KOG, Eukaryotic Orthologous Groups, GEO, Gene Expression Omnibus, PC, phosphatidylcholine, PI, phosphatidylinositol, PG, phosphatidyl glycerol, PE, cephalin, SM, sphingomyelin, VLCFA, very long chain fatty acid, TMT, Tandem Mass Tag, DEPs, differentially expressed proteins.
