# Supplementary material for: Multi-Omics Analysis of Fatty Acid Metabolism in Thyroid Carcinoma
Source: Front Oncol. 2021 Dec 16;11:737127. doi: 10.3389/fonc.2021.737127 (PMC8717782; doi:10.3389/fonc.2021.737127)
Supplement: Supplementary file 1 [file DataSheet_1.docx]

**Supplementary materials**

**Multi-omics analysis of fatty acid metabolism in thyroid carcinoma**

Jinghui Lu, Yankun Zhang, Min Sun, Changyuan Ding, Lei Zhang, Youzi Kong, Meng Cai, Paolo Miccoli, Chunhong Ma, Xuetian Yue

**Supplementary figures**

**Supplementary Fig. 1**


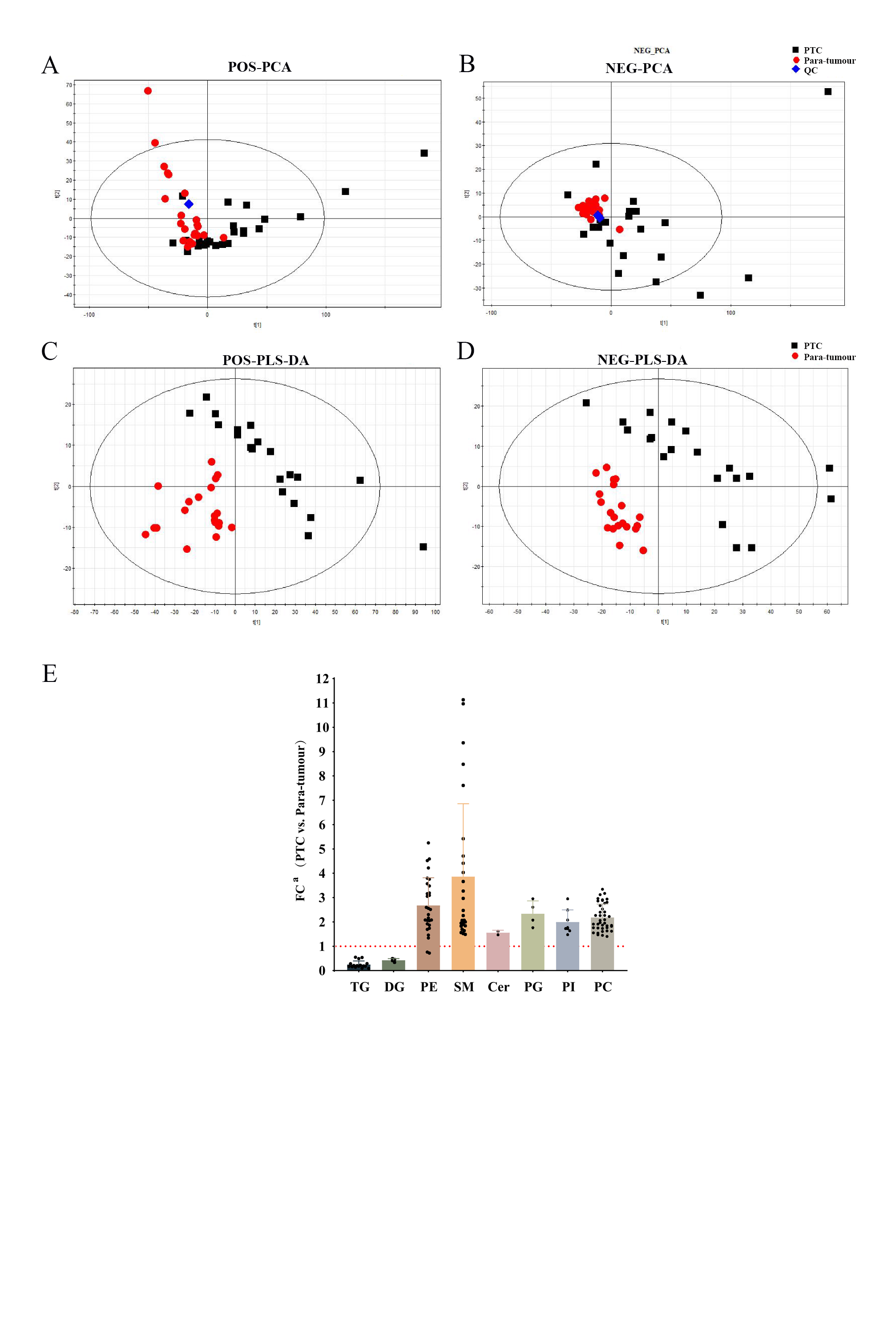


**Supplementary Fig. 1** PCA and OPLS-DA score plot for lipidomic analysis, and differential lipid contents in PTC and para-tumour specimens. (A-D) PCA and OPLS-DA score plot for lipidomic analysis of all samples. PCA results of the quality control sample indicated good system stability. (E) The triglyceride (TG) and diacylglycerol (DG) levels were decreased in the PTC group, and the cephalin (PE), sphingomyelin (SM), Cer, phosphatidyl glycerol (PG), phosphatidylinositol (PI), and phosphatidylcholine (PC) levels were increased in the para-tumour group. Fold change (FC) in PTC/para-tumour specimens.

**Supplementary Fig. 2**


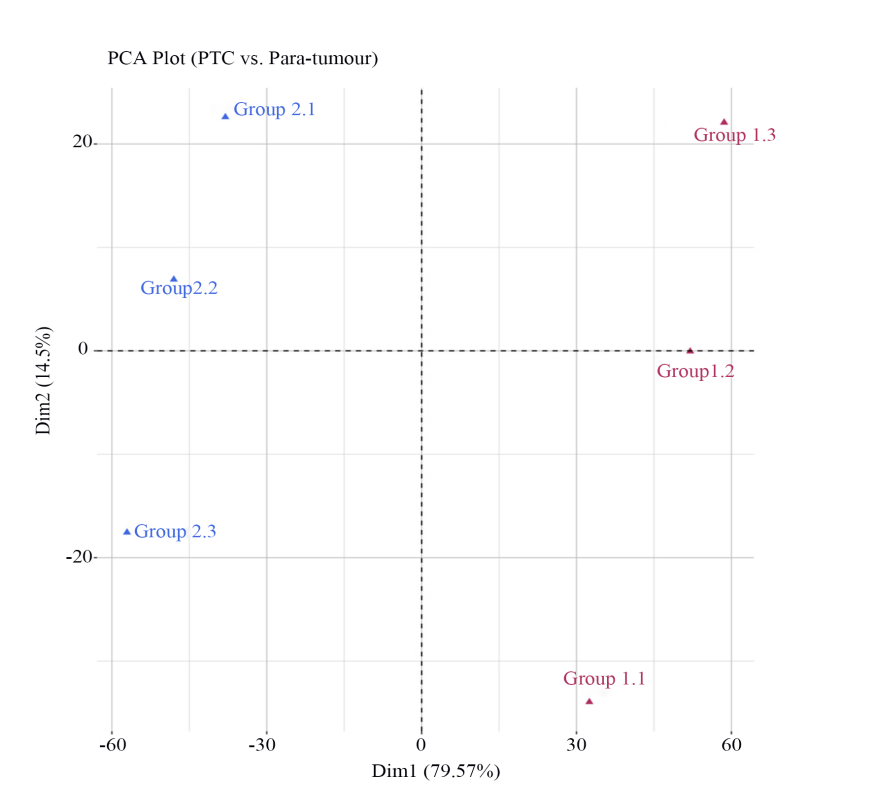


A


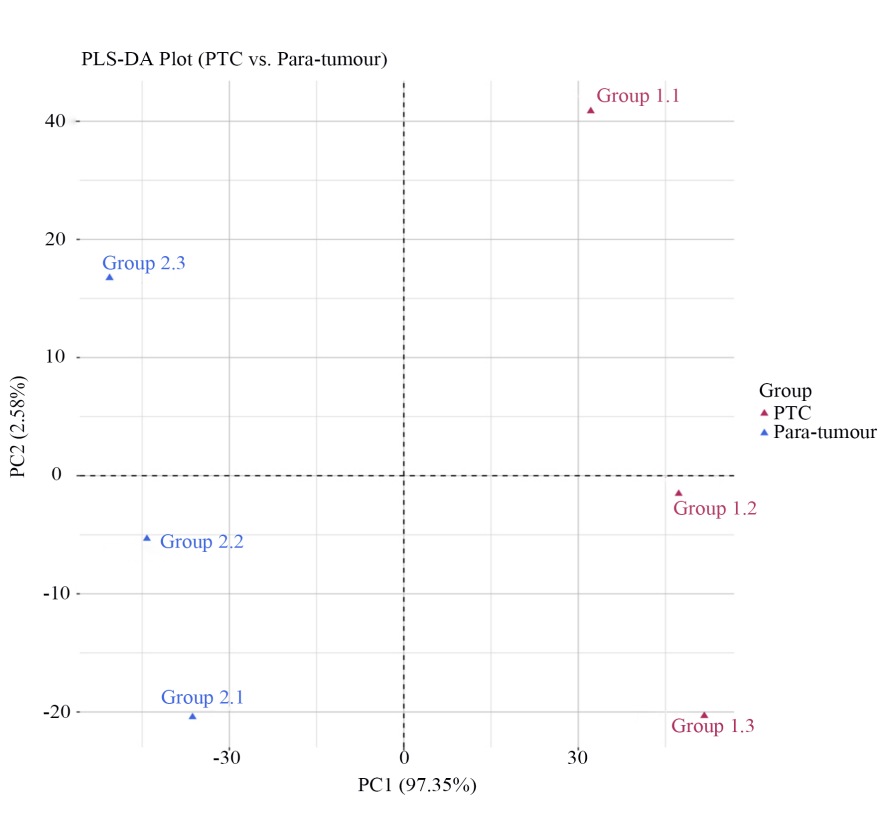


B

**Supplementary Fig. 2** PCA and PLS-DA for proteomic analysis. (A-B) PCA and OPLS-DA score plot for proteomic analysis showing a significant difference between the PTC and para-tumour groups.

**Supplementary Fig. 3**


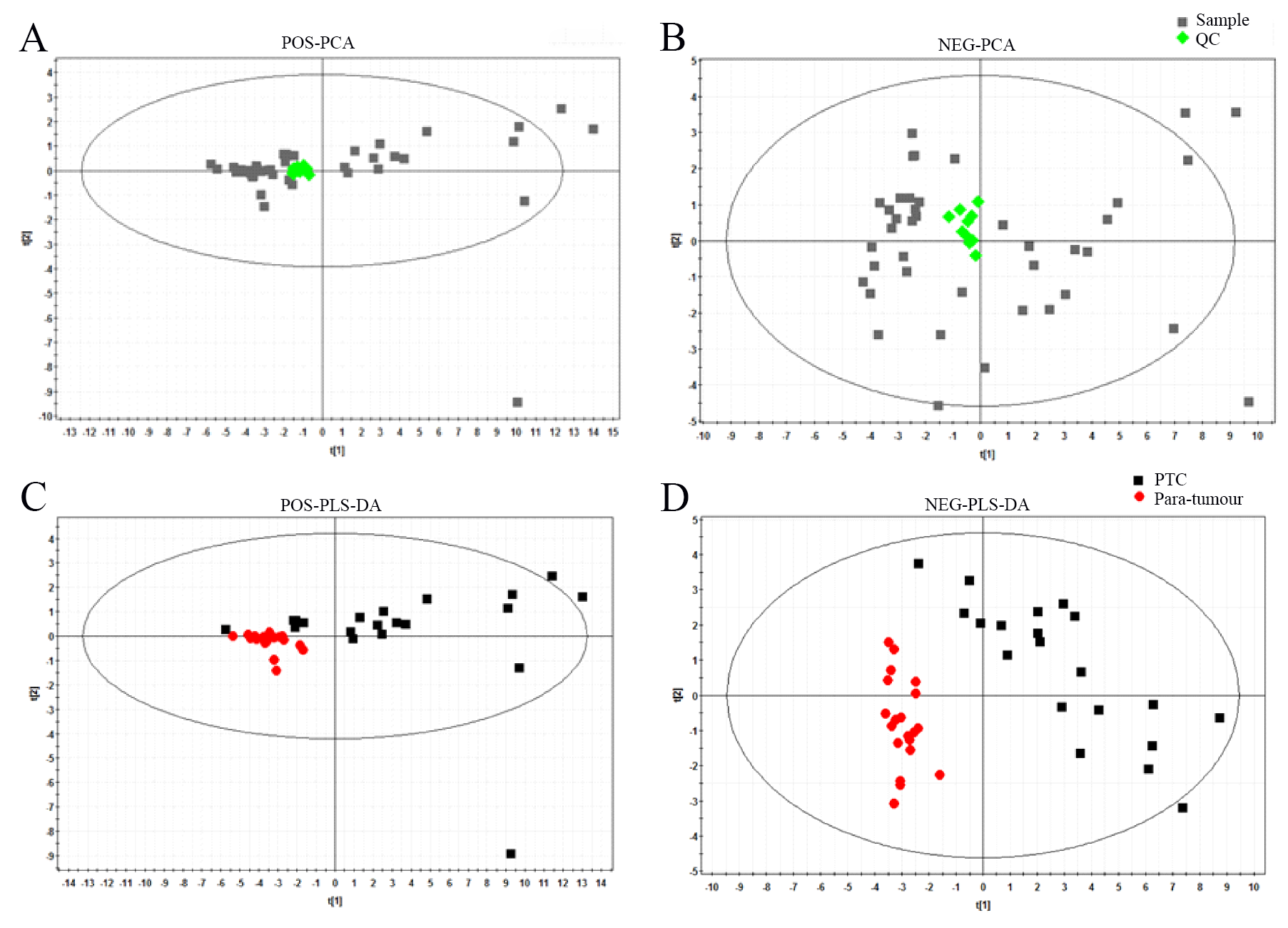


**Supplementary Fig. 3** PCA and PLS-DA for metabolomic analysis. (A-D) The PTC and para-tumour groups could be completely distinguished based on the PCA and PLS-DA results. The quality control samples were well clustered in the positive and negative ion modes, indicating good system stability.

**Supplementary Fig. 4**

B

A


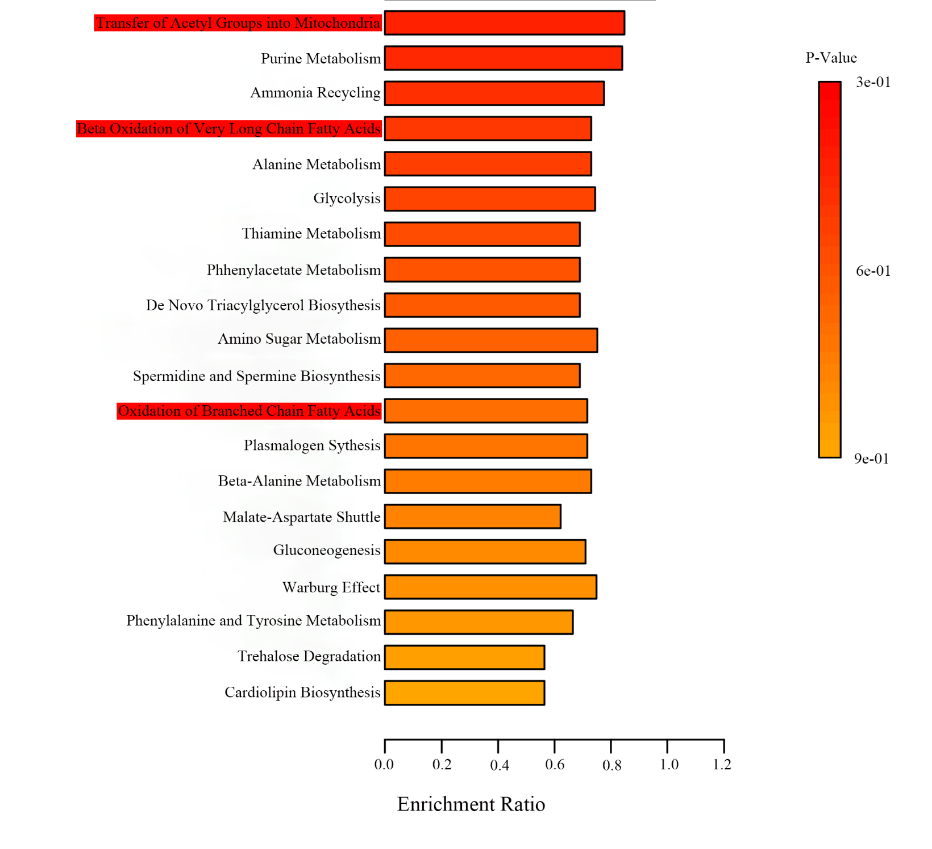

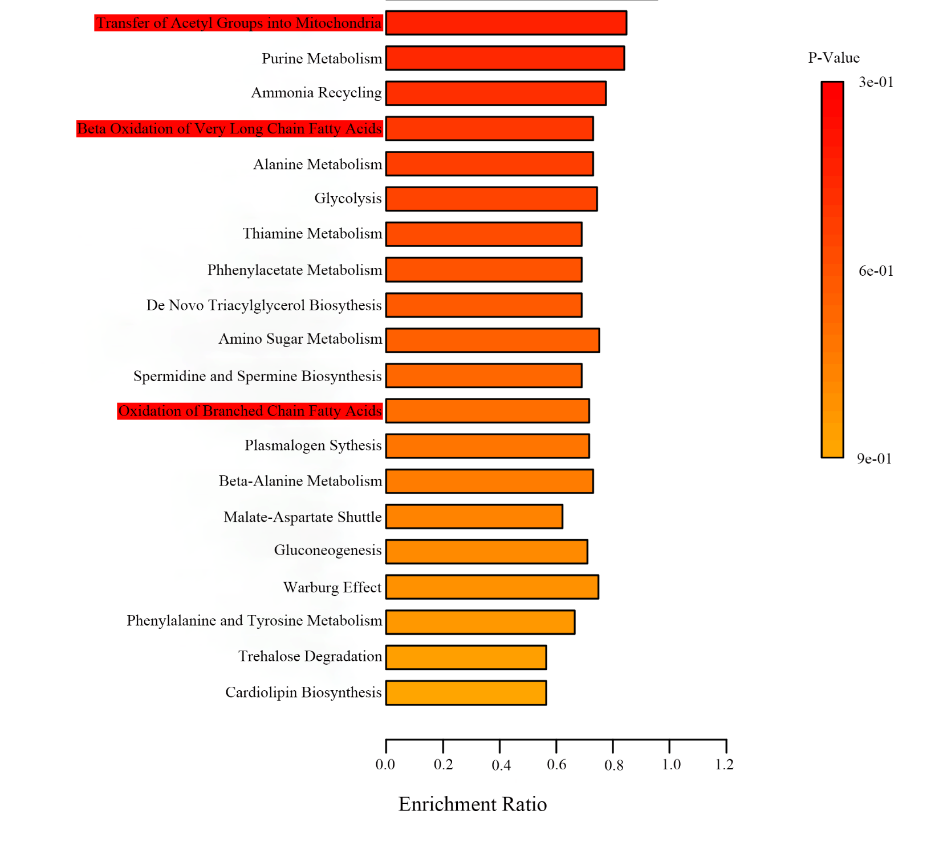


Group

FABP5

FABP4

CD36

ACS

ACAA2

HADH

HADHB

HADHA

CPT2

ACADVL

FATP2

CPT1A

FATP1

MCAT

FATP6

ACADL


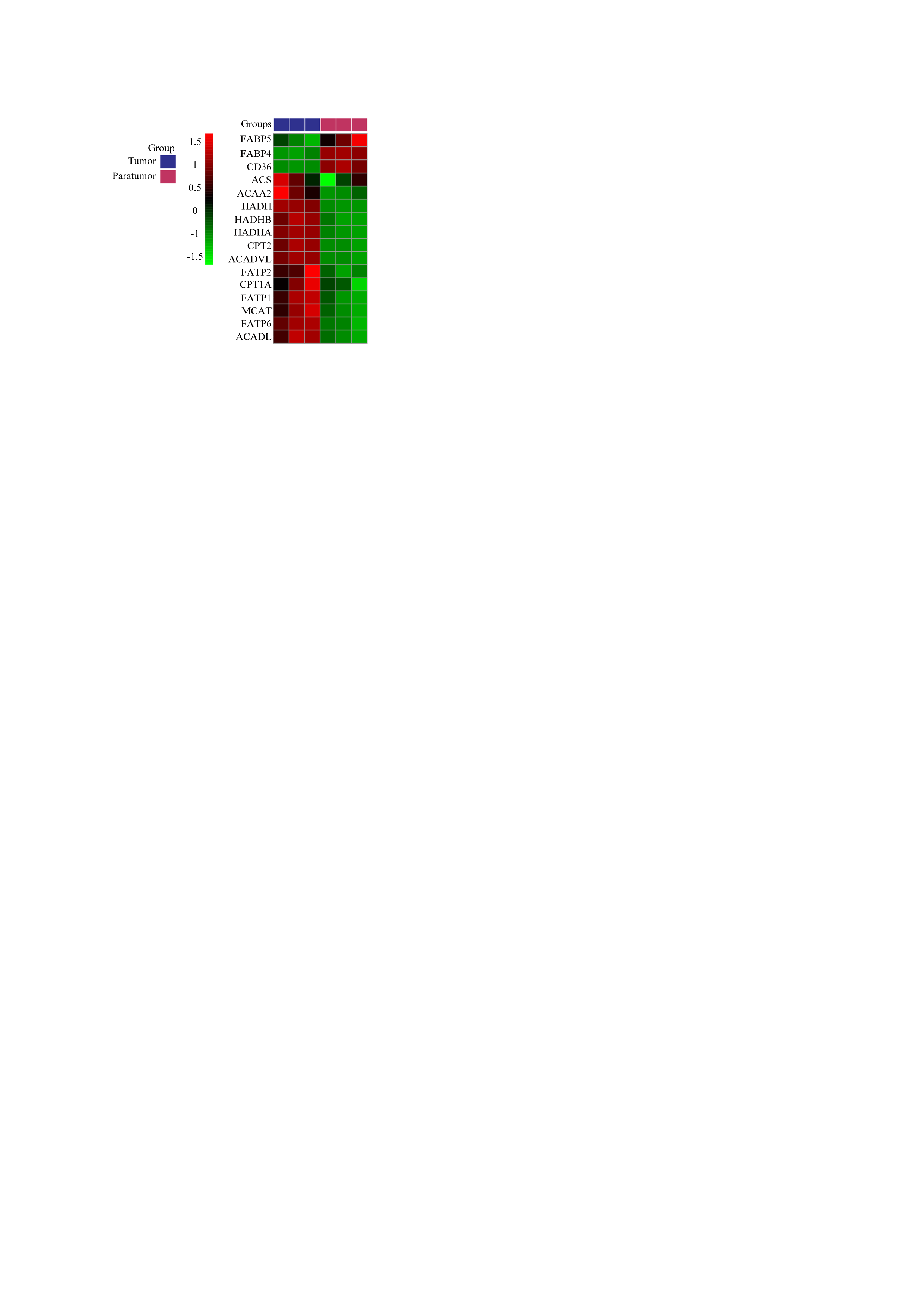

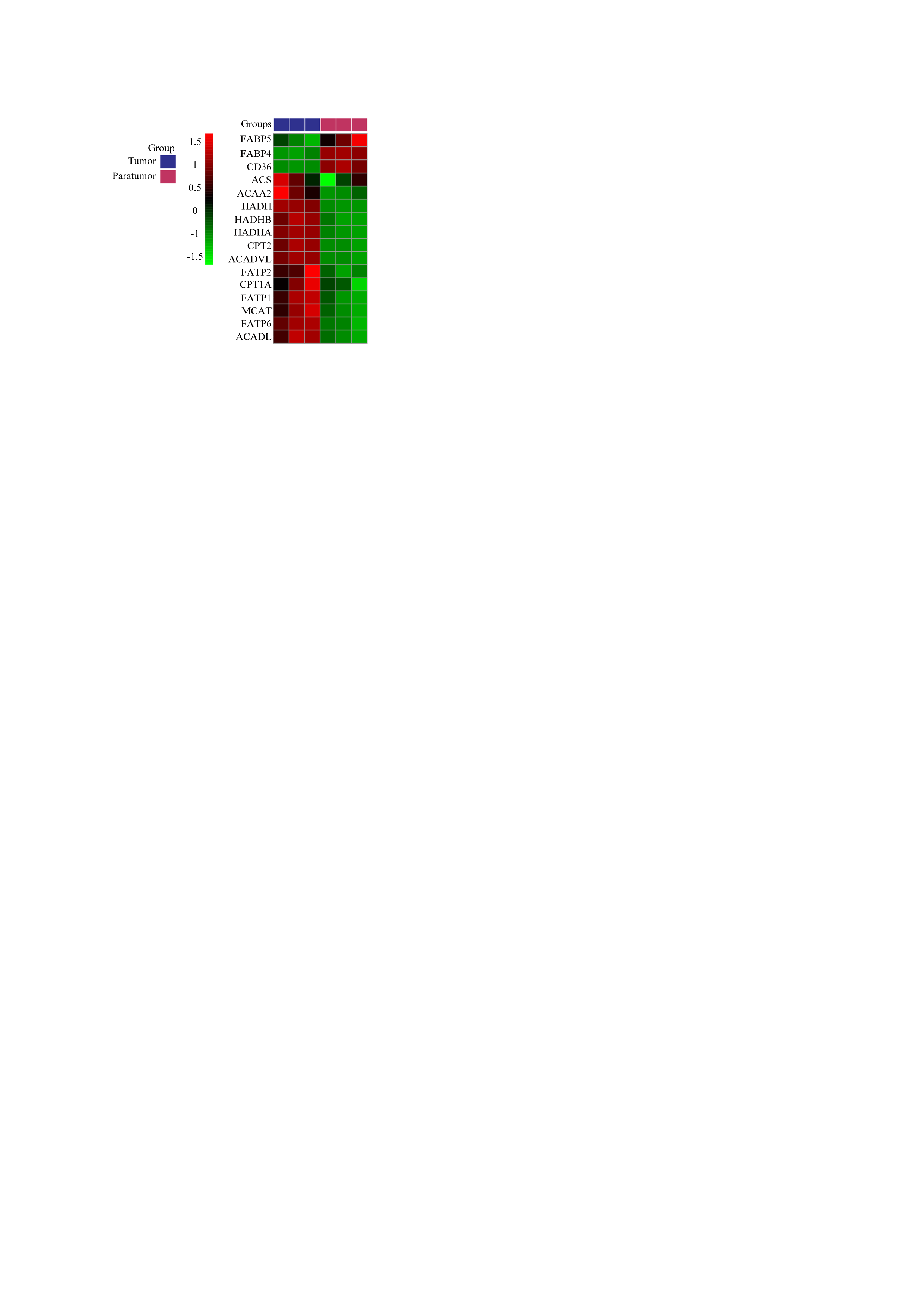


**Supplementary Fig. 4** (A) The pathways indicated in red are associated with fatty acid metabolism and showed high levels of lipid utilisation. (B) Heatmap displaying the distinct lipid metabolism-related proteins in proteomic analysis. Purple legends represent the tumour group, pink legends represent the para-tumour group. Each group includes three samples.

**Supplementary methods**

**Lipidomics**

Ten microliters of each sample were mixed and used it as quality control (QC) sample to monitor the stability of the instrument during operation. LC-MS analysis was performed using Vanquish UPLC and Q Exactive plus mass spectrometer (Thermo Fisher Scientific, Waltham, MA). Waters ACQUITY UPLC BEH C18 (100*2.1mm, 1.7μm) was used for chromatographic separation with 60℃. Mobile phase A is acetonitrile: water (60:40, v/v), mobile phase B is acetonitrile: isopropanol (10:90, v/v), both containing 10 mM ammonium formate. The chromatographic gradient is as follows, 0-0.5min: 5%B, 0.5-2min: 5%-43%B, 2-2.1min: 43%-52%B, 2.1-8.5min: 52%-53%B, 8.5-8.6min: 53%-75%B, 8.6-14min: 75%-90%B, 14-14.5min: 90%-100%B, 14.5-15.5min: 100%B, 15.5-15.7min: 100%- 5%B, 15.7-18min: 5%B. The flow rate is 0.4ml/min, and the injection volume is 5ul. Heated electrospray ionization (HESI) positive and negative ion modes were used for detection. The optimized parameters are as follows, heater temp, 320℃ in positive mode and 300℃ in negative mode, sheath gas flow rate: 45arb, aux gas flow rate: 15arb, sweep gas flow rate: 1arb, Spray voltage: 3.2kV in positive mode and 2.9 kv in negative mode, capillary temp was 320℃, Slens RF level was 50%. MS1 scan ranges was m/z135-2000.

**Proteomics**

Samples were analyzed by Thermo Scientific™ Q Exactive™ Plus Hybrid Quadrupole-Orbitrap™ Mass Spectrometer (Thermo Fisher Scientific, Waltham, MA). The sample pretreatment of proteomics is mainly divided into sample preparation, protein digestion, TMT labeling and high pH reverse phase separation. The sample re-dissolved in water was analyzed by on-line nanospray LC-MS/MS on a Q Exactive HF coupled to an EASY-nano-LC 1200 system (Thermo Fisher Scientific, MA, USA). 3μl peptide was loaded (analytical column: Acclaim PepMap C18, 75μm x 20 cm) and separated with a 90 min linear gradient, from 5% B (B: 0.1% formic acid in ACN) to 30% B. The column flow rate was maintained at 300nl/min with the column temperature of 40°C. The electrospray voltage of 2kv versus the inlet of the mass spectrometer was used. The mass spectrometer was run under data dependent acquisition mode, and automatically switched between MS and MS/MS mode. The parameters were: (1) MS: scan range (m/z) =350–1800, resolution=60,000, AGC target=3e6, maximum injection time=50ms, include charge states=2-7, dynamic exclusion time=30s. (2) HCD-MS/MS: resolution=30,000, isolation window=2, AGC target=1e5, maximum injection time=100ms, collision energy=32. Tandem mass spectra were processed by PEAKS Online (Bioinformatics Solutions Inc., Waterloo, Canada). PEAKS DB was set up to search the SP_Homosapiens_201907.fasta assuming trypsin as the digestion enzyme. PEAKS DB were searched with a fragment ion mass tolerance of 0.02Da and a parent ion tolerance of 15ppm. Carbamidomethylation (C) and TMT 6plex (K, N-term) 229.16 were specified as the fixed modification. Oxidation (M), Deamidation (NQ) and Acetylation (Protein N-term) were specified as the variable modifications. Peptides were filter by 1% FDR and 1 unique.

**Metabolomics**

Ten microliters of each sample were mixed and used it as quality control (QC) sample to monitor the stability of the instrument during operation. UPLC-MS analysis was performed using Waters Xevo TQ-XS Triple Quadrupole Mass Spectrometry (Waters, Milford, MA). Waters ACQUITY UPLC®HSS T3 (2.1×100mm, 2.5um, Waters, Milford, MA) was used for chromatographic separation at 25℃. The mobile phase was 0.1% formic acid solution(A) and ACN with 0.1% formic acid (B). The flow rate was 0.35mL/min and the injection volume were 2ul in positive mode and 4ul in negative mode. Optimized chromatographic gradient was: 0-1min, 5%B, 1-8.5min, 5–95% B, 8.5-10min, 95%B. Post time is set to 3min for re-equilibrate the system. Mass spectrometry was operated in positive and negative modes. The optimized parameters are as follows, capillary voltage, 4kV in positive mode and 3.5kV in negative mode, drying gas flow: 11L/min, gas temperature: 350℃, nebulizer pressure: 45psig, fragmentor voltage: 120 V, skimmer voltage, 60 V. Mass spectrometry acquisition range was m/z 100-1100.
